# Effect of Adhesive Bonding Process Parameters on the Joint Quality of the Middle Layer in Floorboards

**DOI:** 10.3390/ma18204674

**Published:** 2025-10-11

**Authors:** Agnieszka Kujawińska, Michał Rogalewicz, Magdalena Hryb, Krzysztof Żywicki

**Affiliations:** Faculty of Mechanical Engineering, Poznan University of Technology, 3 Piotrowo St., 60-965 Poznan, Poland; michal.rogalewicz@put.poznan.pl (M.R.); magdalena.hryb@put.poznan.pl (M.H.); krzysztof.zywicki@put.poznan.pl (K.Ż.)

**Keywords:** production engineering, wood flooring, adhesives, PVAC, PUR, material efficiency

## Abstract

The quality and durability of adhesive joints in wood flooring are determined by both the type of adhesive and the parameters of the bonding process. This study examines the effects of pressing time and seasoning time on the bending strength of adhesive joints in the middle layer of floorboards manufactured using innovative block-bonding technology. Experimental trials were conducted with two adhesive systems—polyvinyl acetate (PVAC) and polyurethane (PUR)—using a full factorial design and statistical evaluation of joint strength in terms of pressing time and seasoning time. For PVAC, an overall tendency toward increased strength with extended pressing time was observed; however, the strongest effects were associated with interactions between pressing and seasoning times, with the most favorable results obtained for short pressing (5 min) combined with extended seasoning (5 h). In the case of PUR, the relationships were non-linear, and the only statistically significant factor was the interaction between pressing and seasoning times, confirming the necessity of joint optimization. The findings demonstrate that simple one-factor analyses are insufficient to explain adhesive performance, as non-linear and interaction effects are critical in defining joint strength. The results provide new insights for optimizing bonding processes in floorboard production, supporting improvements in material efficiency and mechanical reliability of wood flooring.

## 1. Introduction

The consistently high interest in the use of renewable natural resources in the building and finishing materials industry stems from their favorable technical, aesthetic, and environmental properties. Wood is one such material which, owing to its advantages, continues to be widely used in the production of flooring elements. A representative example of such wood-based products is the three-layer floorboard.

**Figure 1:**
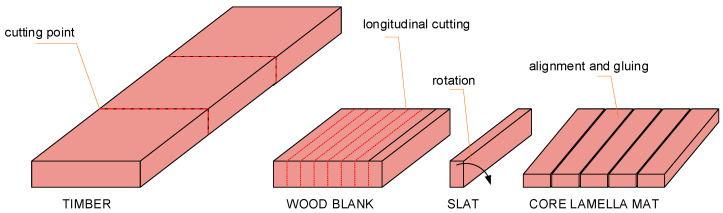
Outputs of the subsequent stages of the middle layer manufacturing process [9].

A typical three-layer floorboard consists of a top layer (usually made of noble wood), a middle layer (serving as the load-bearing component), and a bottom layer (compensating for deformations). The middle layer of the floorboard, commonly referred to as Transverse–Axial Formation (TAF), represents one of its key structural elements, as it determines dimensional stability and resistance to changes in ambient humidity [1,2]. In wood flooring construction, cores made of high-density fiberboard, plywood, or softwood (e.g., pine) are commonly used [3]. Traditionally, the middle layer is made of thin softwood slats arranged perpendicularly to the grain direction of the top layer. These slats are selected, graded, and glued into mats (TAF), which are subsequently combined with the remaining layers in the form of blocks from which the final product is manufactured [4]. The production process of TAF involves several key stages: raw material preparation (sawn timber → wood blanks → slats) (Figure 1), quality control (visual inspection), and slat gluing to core lamella mat [4,5].

The mats are bonded with the top and bottom layers of the floorboard using adhesive. The assembled block undergoes further processing (i.e., profiling of locking joints, varnishing) and constitutes the final product—a three-layer floorboard.

In the manufacturing process of the TAF mat, the key challenge is to maintain its geometry within tolerance limits. Dimensional instability of TAF may result from the insufficient stability of the input material-sawn timber. The main cause of excessive dimensional variability in sawn timber lies in the natural heterogeneity of wood as a raw material, as well as in the sawing and drying processes, which are largely unpredictable [6,7]. The literature indicates that shifts in the geometry of sawn timber toward tolerance limits result in an increase in material waste in the TAF production process by an average of 15%–20% [8]. In the operation of cutting blanks into slats, the primary cause of material losses—reaching up to 30%—is insufficient planning of the slats. Such defects occur as a consequence of improper positioning of the blanks [9]. The combined effect of these factors ultimately leads to a reduction in process capability, an increase in material losses, a limitation of dimensional repeatability of the elements, and a higher risk of producing semi-finished components that do not meet structural and assembly requirements.

According to the literature [10,11,12,13,14,15,16,17,18,19], proposals for improving raw material efficiency in the manufacturing of the middle layer include replacing solid wood with alternative materials or adopting alternative strategies for TAF production. In the first approach, which involves the substitution of wood with other materials in the construction of the floorboard’s middle layer, several alternatives have been identified. These include particleboards and fiberboards, such as oriented strand board and medium-density fiberboard. The use of such wood-based materials improves dimensional stability and lowers material costs. Studies indicate that these structures are widely employed as cores in flooring elements due to their predictable mechanical properties [10,11,12]. The main limitation of applying these materials as the middle layer in floorboards lies in their low resistance to ambient humidity, combined with a susceptibility to edge swelling and delamination under elevated humidity conditions. Such behavior may compromise joint integrity and induce surface layer deformations, which is particularly critical for floors installed by permanent gluing or in floating systems. An alternative solution discussed in the literature involves the use of composites, most frequently wood–polymer systems [11,12,13,14,15,16]. Composites exhibit high resistance to ambient humidity; however, life cycle assessment reveals considerable environmental burdens associated with their production, use, and end-of-life management. For instance, the carbon footprint of wood panel composite boards made with PVC and wood flour amounts to approximately 145 kg CO_2_e/m^2^, which can be reduced to around 65 kg CO_2_e/m^2^ through material and energy optimization strategies [17]. In comparison, wood flooring exhibits a much lower carbon footprint, around 39.3 kg CO_2_e/m^2^, dropping to 16.4 kg CO_2_e/m^2^ when biogenic carbon storage is taken into account [18,19].

The second approach, focusing on alternative strategies for TAF manufacturing, introduces novel technologies, frequently relying on adjustments to the sequence of operations in the production process [14]. One example is a technology involving the pre-gluing of sawn timber into larger elements, which are subsequently cut into smaller blocks used to form the final TAF mat. This approach has been investigated, among others, by the authors as part of the BIOSTRATEG2/298950 project carried out by the Faculty of Mechanical Engineering, Poznan University of Technology, in cooperation with Barlinek Inwestycje Sp. z o.o. [20].

In the wood industry, urea–formaldehyde, phenol–formaldehyde, melamine–urea–formaldehyde, and polyurethane (PUR) adhesives are commonly used, with an increasing interest in bio-based adhesives produced mainly from soy, lignin, or tannins. According to the literature, both the chemical composition of the adhesive and the parameters of the bonding process, including the method of adhesive application, pressing pressure, and pressing time, are of crucial importance for the quality of the product [21,22]. Studies on floorboards bonded with PUR adhesives have shown that the modulus of elasticity and bending strength increase with higher pressure and adhesive quantity, while pressing time exhibits a non-linear effect—pressing that is too short results in insufficient bonding, whereas excessive pressing time promotes local inhomogeneities [22]. In the case of bonding wood-based materials such as oriented strand board, the development of interlayer stresses and the tendency to cupping arise not only from the anisotropy of wood but also from the permeability and stiffening effect of the bond layer. Three-dimensional numerical simulations have shown that the selection of adhesive and material properties significantly alters the stress distribution in regions near free edges and affects the material response under humidity cycling, which in turn influences adhesive joint durability and the risk of surface cracking [10,21].

Blanchet et al. conducted comparative tests of four adhesive classes subjected to accelerated aging under hygrothermal cycling (summer–winter): phenol–formaldehyde, urea–formaldehyde, melamine–urea–formaldehyde, and polyurethane. Polyurethane adhesives maintained the highest and most stable shear strength of the adhesive joint, whereas urea–formaldehyde and melamine–urea–formaldehyde exhibited a gradual decrease in load-bearing capacity with the number of cycles. These findings are consistent with industrial observations regarding the resistance of PUR adhesive joints to variable environmental conditions [23].

Bomba et al. examined the dependence of the strength increase in a bonded joint on the curing time for various types of polyvinyl acetate (PVAC) adhesives [24]. On the other hand, Kowluk and Fuczek studied bending strength of PVAC adhesive in comparison with urea–formaldehyde resin adhesive for particleboards, and proved that it is lower on average [25].

Lee and Kim reported that the type of adhesive exerts a significant effect on the load-bearing performance of timber structures. Systems bonded with phenol–formaldehyde adhesives achieved higher compressive strength values than their counterparts manufactured with PUR adhesives. For applications requiring maximum load-bearing capacity, these results highlight the necessity of a compromise in adhesive selection, taking into account both the durability of adhesive joints under variable environmental conditions and the achievement of optimal mechanical properties [21].

Scientific studies provide evidence that bonding process parameters are key factors shaping the mechanical properties and strength of wooden elements. At the same time, there is a lack of comprehensive analyses defining the optimal selection of adhesive type and bonding process parameters in relation to this TAF manufacturing technology. This represents an important research gap, as bonding parameters, such as adhesive type, application rate, pressing time, and temperature, affect not only the mechanical performance and dimensional stability of the middle layer of floorboards but also their service durability and susceptibility to deformation under variable environmental conditions. The lack of knowledge in this area hinders process optimization and may lead to underutilization of the full potential of this technology in the flooring industry.

The presented research results constitute part of the research and development project which aimed at improvement of raw wood efficiency in the industrial production processes mentioned earlier. The main objective of the research was to increase material efficiency in the production of flooring elements. The key original solution was to bond boards into larger blocks after the drying process, which were then cut into modules, instead of—as in traditional approaches—into individual slats intended for the middle layer. The research was directed toward designing an innovative wood bonding process, and addresses the lack of analysis regarding the selection of adhesive type and the bonding process parameters in this process. The following sections present the results of experimental studies on bonding processes, aimed at evaluating the strength of adhesive joints in the context of their application in the innovative technology of manufacturing the middle layer of floorboards. The aim of the experiment was also to determine which bonding process parameters are statistically significant, the interaction between them, the nature of their variability, and the optimal setting for them.

## 2. Materials and Methods

**Figure 2:**
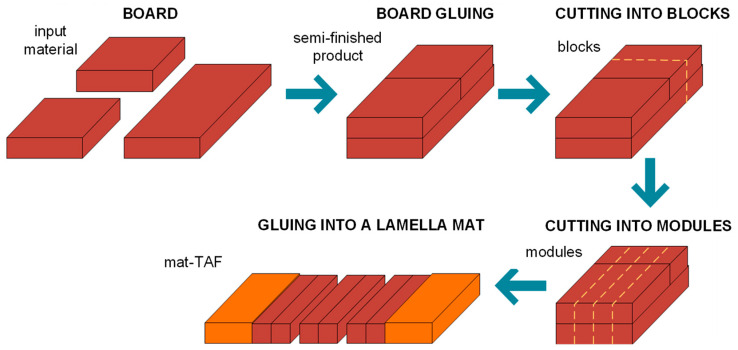
Conceptual framework of the new process for manufacturing the middle layer of a floorboard.

The core of the new technology consists of bonding boards into packages following the drying operation, which are subsequently cut into modules instead of individual slats (Figure 2).

**Figure 3:**
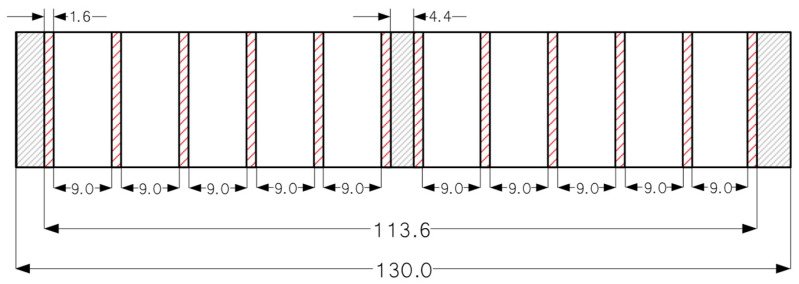
Diagram of cutting the bonded block into test samples.

On the basis of previous research results [20], experimental trials were designed to determine the appropriate adhesive type together with pressing and seasoning times, while maintaining the required bond quality. Within these trials, two sawn timber boards (pine wood) with dimensions 2950 mm (length) × 130 mm (width) × 26 mm (thickness) were bonded (Figure 3).

**Figure 4:**
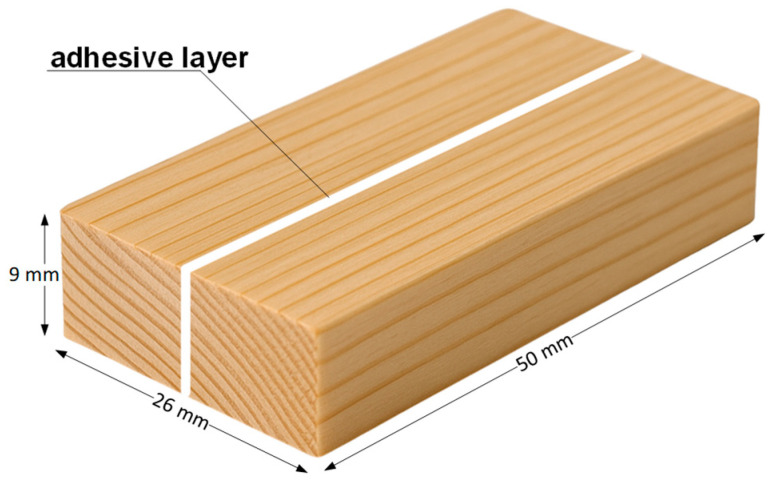
Exemplary sample for testing.

The property examined was the bending strength of the adhesive joint. Mechanical test specimens were prepared with dimensions of 9 × 26 × 50 mm (thickness × width × length) (Figure 4).

The specimens were prepared in such a way that growth rings were arranged horizontally in relation to the module surface (Figure 4), ensuring that the forces generated during loading acted in the tangential direction. For testing, specimens with a similar width of annual growth rings were selected, free from material defects such as knots, resin pockets, cracks, machining flaws, or visible delamination in the adhesive joint area. The wood fibers were oriented parallel to the specimen edges (Figure 4). The adhesive was applied to the sawn timber with a roller across its entire surface.

**Figure 5:**
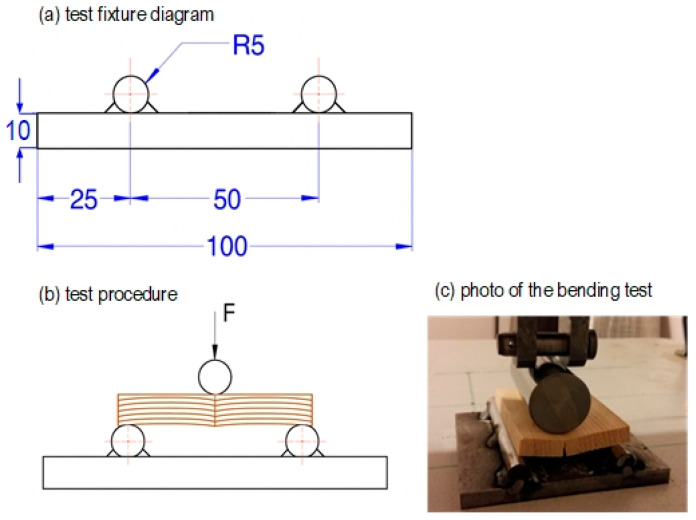
(**a**) Diagram of the testing stand. (**b**) Positioning of the specimen in the stand. (**c**) Photo of the test.

The experiment was carried out using a testing stand for bending force determination (Figure 5), which reflects bonding strength. The setup consisted of two cylindrical supports and a cylindrical loading head that applied force in the middle of the specimen. The fixture, prepared and constructed by Barlinek Inwestycje Sp. z o.o. (1 Przemysłowa St., Barlinek, Poland), was mounted on a Zwick 100 universal testing machine, which continuously recorded the applied force until specimen failure. The tests were performed at a constant crosshead speed of 5 mm/min (millimeters per minute; hereafter, “min” denotes minutes).

Two types of adhesives were used in the experiments: PVAC and PUR—they were provided by Barlinek Inwestycje Sp. z o.o. The effects of pressing time and seasoning time (here understood as the static curing time after bonding) on bending strength (determined by the measured force) were investigated. A full factorial design was applied, considering two factors at three levels each. The experimental designs for PVAC and PUR are presented in Table 1. Each experiment was replicated ten times.

Differences in pressing times between PVAC and PUR adhesives stem from their distinct bonding mechanisms and manufacturer specifications, which define the minimum technological requirements for achieving comparable adhesive joints maturity. PVAC (dispersion) adhesives attain initial strength primarily through water evaporation and penetration into the substrate, making short pressing times (5–15 min) sufficient to stabilize the joint. In contrast, PUR (reactive) adhesives cure by reacting with ambient moisture, with cross-linking kinetics governed by water diffusion and temperature; consequently, longer pressing times (20–40 min) are required to maintain element contact until a continuous PUR structure is established. Pressing-time levels for both systems were determined based on technical data sheets, manufacturer guidelines (open time, press time, minimum loading time), as well as material (wood species, moisture content) and environmental factors, to ensure repeatability, prevent premature adhesive joint loading, and to enable reliable assessment of process parameters on bending strength. Seasoning times (1–5 h) were selected to facilitate bending strength comparison at consistent intervals after bonding.

The specimens were subjected to bending tests, and the results were statistically analyzed using box plots, descriptive statistics, and plots associated with the methodology of experimental design and analysis.

## 3. Results 

### 3.1. PVAC Adhesive

**Figure 6:**
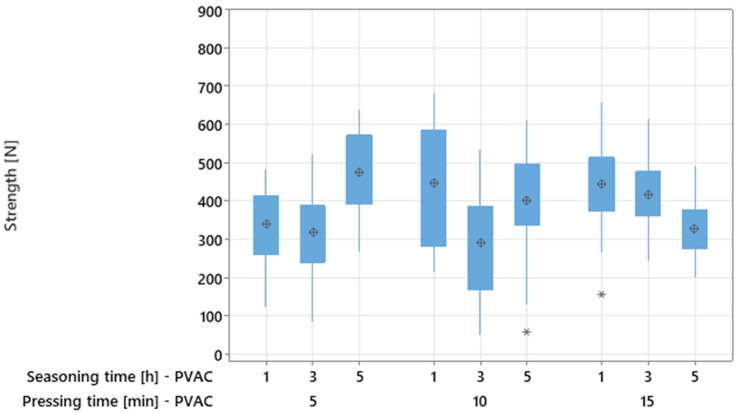
Boxplots for factorial experiment for PVAC (asterisks indicate outliers and are located more than 1.5 IQR (interquartile range) from the edges of the boxes).

Active experiment No. 1 examined the effects of pressing time and seasoning time on the bending strength of PVAC-bonded adhesive joints. Preliminary analysis using box plots (Figure 6) revealed no consistent trends demonstrating a pronounced influence of either factor. For both pressing time and seasoning time, substantial variability in strength values was observed, while the median values remained comparable (Figure 6).

The analysis of descriptive statistics for individual levels of pressing time showed a gradual increase in the mean strength with increasing values of this factor—from 377.06 N at 5 min, to 389.71 N at 10 min, and 398.88 N at 15 min (Table 2).

**Figure 7:**
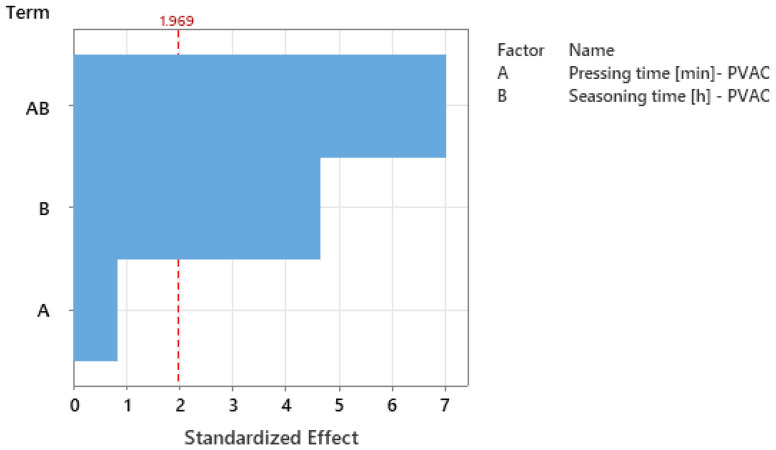
Pareto chart of the standardized effects for PVAC experiment: response–strength [N]; significance level α = 0.05.

The Pareto chart of standardized effects (Figure 7) indicates that, in the PVAC adhesive experiment, the interaction between pressing time (A) and seasoning time (B) had the strongest effect on bending strength.

The standardized effect exceeds the statistical significance threshold (critical value ≈ 1.969, α = 0.05), indicating that the influence of one factor on strength is strongly conditioned by the level of the other. The next significant factor is seasoning time (B), whose effect also surpasses the threshold, confirming its clear and direct impact on strength. In contrast, pressing time (A) remains below the significance level, suggesting only a minor direct effect compared to the interaction and seasoning time.

**Figure 8:**
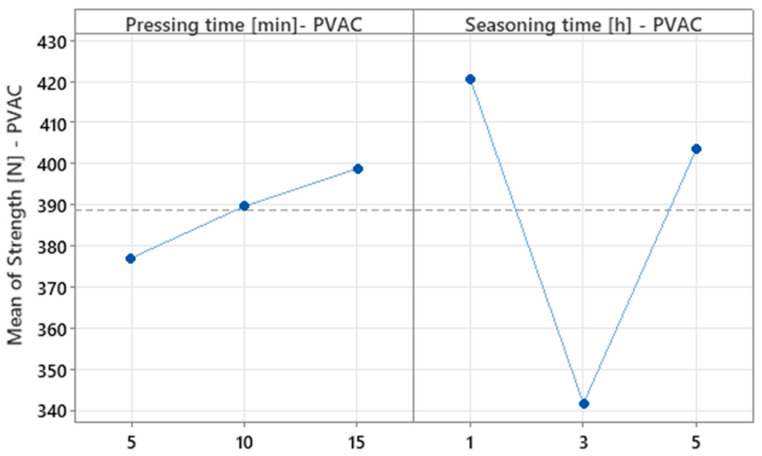
Main effects plot for strength for PVAC experiment.

The main effects plot (Figure 8) supports the initial finding that mean strength increases progressively with pressing time. For seasoning time, however, the trend is less distinct, and the line pattern suggests that although this factor is statistically significant, the relationship between seasoning time and strength is not monotonic.

**Figure 9:**
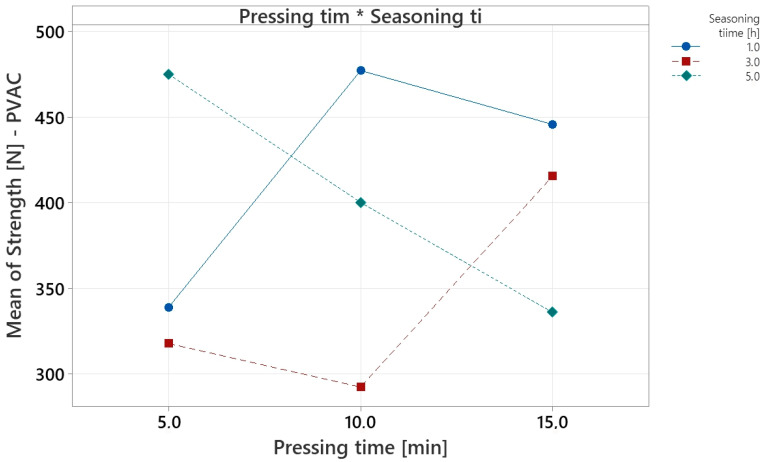
Interaction plot for PVAC experiment.

The interaction plot (Figure 9) shows that the influence of pressing time on strength is conditioned by the level of seasoning time, confirming the interaction between the studied factors (Figure 7).

With a seasoning time of 1 h, changes in pressing time produced only moderate variations in strength, suggesting that such a short curing period restricts the full development of mechanical properties regardless of pressing time. At 3 h seasoning, the relationship differed: increasing pressing time from 5 to 10 min reduced strength, while extending to 15 min produced a significant increase. At 5 h seasoning, the opposite effect was observed—the highest strength occurred at the shortest pressing time (5 min), whereas longer pressing caused a steady and significant decrease. These findings imply that, in industrial practice, optimal combinations of pressing and seasoning times must be selected, reflecting the mutual influence of both factors on the mechanical performance of adhesive joints.

### 3.2. PUR Adhesive

**Figure 10:**
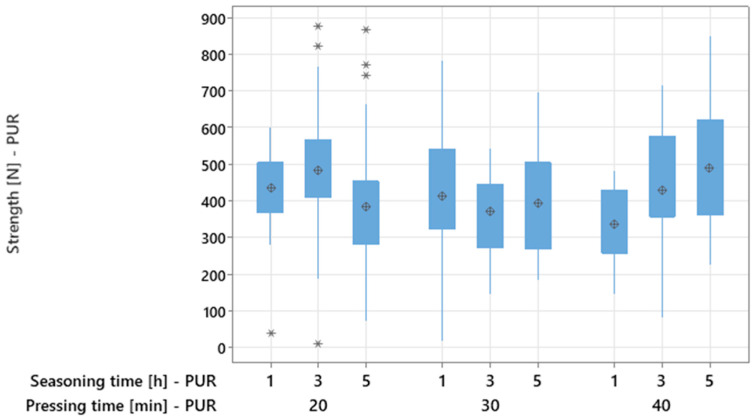
Boxplots for factorial experiment for PUR (asterisks indicate outliers and are located more than 1.5 IQR (interquartile range) from the edges of the boxes).

Active experiment No. 2 examined the effects of pressing and seasoning times on the bending strength of PUR-bonded adhesive joints. Box plots (Figure 10) revealed no monotonic increase in strength with extended pressing or seasoning times. Substantial overlap between distributions and comparable medians across groups were observed. The plots also show heterogeneous scatter (box widths and whisker lengths) and the presence of outliers, both of which restrict firm conclusions.

The descriptive statistics indicate that the mean strength values for PUR joints vary non-linearly with pressing and conditioning times (Table 3). For 20 min pressing, the average was to 433.95 N, with the highest mean (485 N) at 3 h seasoning and the lowest (383 N) at 5 h. At 30 min pressing, the overall mean dropped to 393.01 N. For 40 min pressing, the mean increased to 418.09 N, with the maximum strength (490 N) observed at 5 h seasoning. These results confirm strong variability between means and suggest that extreme combinations of pressing and conditioning times tend to yield higher strength than intermediate levels.

**Figure 11:**
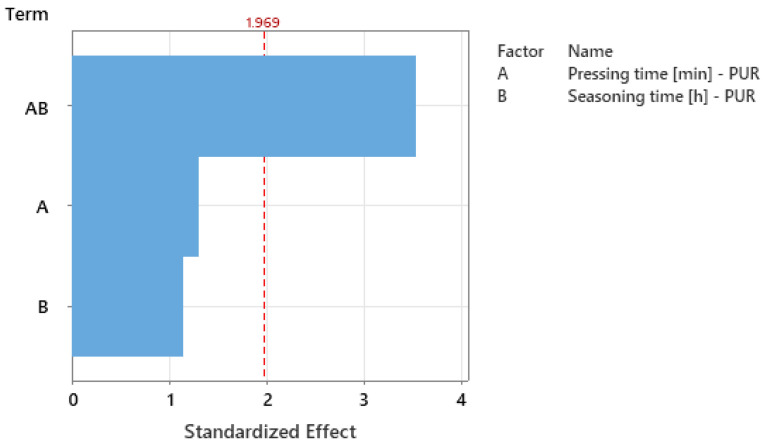
Pareto chart of the standardized effects for PUR experiment: response–strength [N]; significance level α = 0.05.

The analysis of the Pareto chart (Figure 11) of standardized effects (α = 0.05; critical value ≈ 1.969) showed that the only statistically significant effect is the interaction between pressing time (A) and seasoning time (B). Its standardized value is approximately 3.5, exceeding the significance threshold, indicating that the influence of one factor depends on the level of the other. The main effects—pressing time (A) and seasoning time (B)—have values below the significance threshold and therefore do not significantly affect strength within the studied range when considered separately. This indicates that in process optimization, both factors must be selected jointly because their effects are mutually correlated.

**Figure 12:**
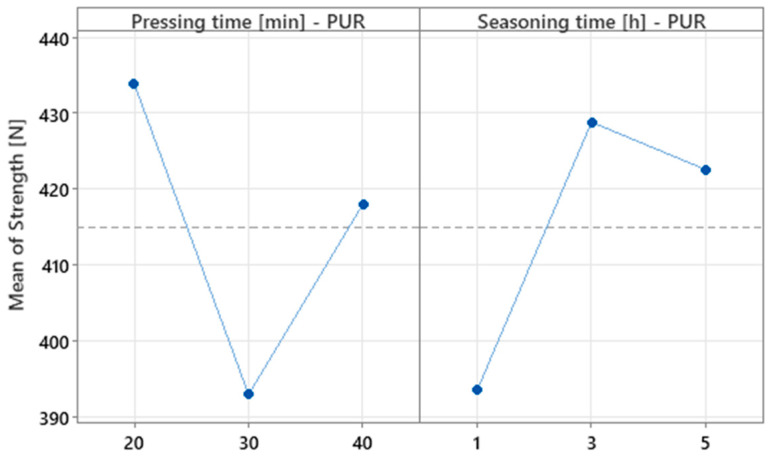
Main effects plot for strength for PUR experiment.

The main effects plot (Figure 12) indicates no linear relationship between factor levels and mean strength. For pressing time, the curve shows a non-linear effect, with reduced mean strength at the intermediate level and higher values at the extremes. Seasoning time exhibits a similarly non-linear pattern. The point distribution confirms the absence of a monotonic trend and suggests an interaction effect, necessitating the joint inclusion of both factors in the model.

**Figure 13:**
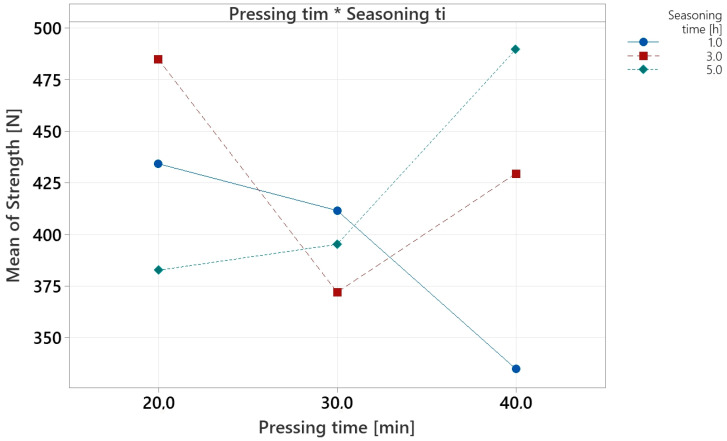
Interaction plot for PUR experiment.

The interaction plot (Figure 13) indicates that the effects of the examined factors are not fully additive. For short seasoning time (1 h), changes in pressing time result in a decrease in strength. For 5 h seasoning, extending pressing time from 20 to 40 min leads to an increase in strength, suggesting a synergistic effect of both factors. The non-parallel lines in the interaction plot confirm the significant nature of the interaction identified in the Pareto analysis.

## 4. Discussion

The experiments with PVAC and PUR adhesives enabled the identification of distinct mechanisms governing the development of adhesive joint strength, dependent upon process parameters. While the statistical analysis confirmed significant interactions between pressing and seasoning times, the observed effects can be explained by the underlying adhesion and cohesion mechanisms, as well as the physicochemical properties of both the adhesives and the wood substrate. Previous studies have emphasized that adhesive penetration into the porous wood structure, the extent of polymerization, and the balance between adhesive cohesion and substrate adhesion are decisive for the final strength of the bond [26,27].

Our findings confirm these observations and provide new insights specific to the bonding of floorboard middle layers. For PVAC adhesive, no monotonic dependence on seasoning time was observed. In our study, the highest mean strength values were achieved after 1 h of seasoning, followed by a decrease at 3 h and a partial recovery at 5 h. This behavior suggests that PVAC bonds stabilize relatively quickly, as water evaporation during the initial curing stage enables polymer chain coalescence and the development of cohesive strength. Similar phenomena have been reported, with most of the strength of PVAC joints being established within the first hours after bonding [28,29]. The limited positive effect of increased pressing time in our experiments indicates that higher pressure improves adhesive penetration and mechanical interlocking but only to a small extent, and excessive pressure may risk starving the joint, a mechanism also discussed in the literature [30]. Importantly, our results demonstrate that favorable performance was not determined by isolated parameter values but by specific combinations—for instance, pressing for 5 min combined with 5 h of seasoning produced the highest strength. This finding highlights the significance of interaction effects and cautions against process optimization based on single-factor analysis.

The behavior of PUR adhesive proved more complex, exhibiting clear non-linear relationships. In our tests, the highest strengths were obtained at both short and long pressing times, while intermediate pressing consistently resulted in reduced strength. This trend can be explained by the dual mechanism of PUR bonding, which relies on both mechanical penetration and chemical reactions between isocyanate groups and wood moisture. Short pressing may allow adequate adhesive flow and initial polymerization, while long pressing ensures complete chemical curing. In contrast, intermediate pressing appears insufficient for either mechanism to dominate, leading to weaker joints. A similar non-linear influence of pressing on PUR bond performance has been confirmed in recent studies [31,32,33]. Moreover, the slight decrease in strength after prolonged seasoning observed in our experiments corresponds with earlier findings that excessive curing may cause embrittlement of the PUR matrix and reduced flexibility at the bond line [34]. These results underline the importance of the interaction between pressing and seasoning times, which was statistically significant in our analysis and is consistent with the reports of synergistic effects in PUR bonding.

A direct comparison of the two adhesives shows that while PVAC relies primarily on physical adhesion and mechanical interlocking, developing most of its strength rapidly, PUR bonding depends on chemical cross-linking and is therefore more sensitive to curing conditions and factor interactions. Our results confirm that in both cases simple linear dependencies are absent, and the strength of the adhesive joint must be interpreted in terms of non-linear, multifactorial interactions. This conclusion aligns with broader research indicating that adhesive bonding in wood cannot be fully described by one-dimensional parameter studies but requires multi-factorial models that capture synergistic effects [35,36].

From a technological perspective, our findings have direct implications for industrial practice. For PVAC, short pressing (5 min) with extended seasoning (5 h) maximized strength, making this configuration suitable for processes prioritizing high joint quality. Alternatively, longer pressing (10–15 min) with shorter seasoning (1 h) provided relatively high strength while enabling shorter production cycles, which may be advantageous in industrial conditions. For PUR adhesives, intermediate pressing times should be avoided, while both short and long pressing, combined with medium or long seasoning, yielded favorable results. Such guidelines are particularly relevant for floorboard production, where bonding efficiency must be balanced with cycle time and production costs.

Overall, our experiments confirm that single-factor analyses are insufficient, and that reliable process recommendations require recognition of non-linear responses and factor interactions. The results are in agreement with international literature emphasizing the complexity of adhesion and cohesion in wood bonding. At the same time, the present study extends these findings by providing experimental evidence on parameter sensitivity specific to floorboard lamella bonding, thereby offering practical guidelines for industrial application. Future research should focus on parameters such as adhesive application method, layer thickness, and distribution uniformity, as these can further influence adhesion mechanisms and the final performance of the bond. The research results are not only applicable to the production of the middle layer of floorboards but may also have broader application, for example, in other wooden or wood-based structures in which the gluing process is used, such as in the furniture industry, window and door joinery, as well as the processing of wood-based composites.

## Figures and Tables

**Table 1:** Experimental plan for PVAC and PUR adhesives.

Experimental Trial	Adhesive Type	Pressing Time [min]	Seasoning Time [h]
No. 1	PVAC	5	1
	PVAC	5	3
	PVAC	5	5
	PVAC	10	1
	PVAC	10	3
	PVAC	10	5
	PVAC	15	1
	PVAC	15	3
	PVAC	15	5
No. 2	PUR	20	1
	PUR	20	3
	PUR	20	5
	PUR	30	1
	PUR	30	3
	PUR	30	5
	PUR	40	1
	PUR	40	3
	PUR	40	5

**Table 2:** Descriptive statistics of PVAC adhesive experiment results.

			Strength [N]—PVAC						
Pressing Time [min]	Seasoning Time [h]	Mean	SE of Mean	StDev	Minimum	Q1	Median	Q3	Maximum
5	1	338.80	17.41	95.376	124.7	259.1	359.5	414.2	483.1
	3	317.63	19.44	106.460	83.2	240.5	290.8	388.4	523.1
	5	474.76	18.65	102.131	267	391.4	471.2	572.37	641.7
mean for 5 min pressing		377.06							
10	1	476.94	24.36	133.444	216.3	383.6	498.4	594.68	682.9
	3	292.23	23.7	129.830	49.4	169.3	335.2	387.5	535.5
	5	399.96	25.87	141.721	56.3	337.1	401.8	496.7	612.3
mean for 10 min pressing		389.71							
15	1	445.51	19.58	107.270	155.5	373.2	455.5	514.1	660.4
	3	415.35	15.67	85.825	243.3	360.6	420.3	477.1	614.3
	5	335.79	12.31	67.446	201.8	286.1	327.2	394.2	492.2
mean for 15 min pressing		398.88							

**Table 3:** Descriptive statistics of PUR adhesive experiment results.

			Strength [N]—PUR						
Pressing Time [min]	Seasoning Time [h]	Mean	SE of Mean	StDev	Minimum	Q1	Median	Q3	Maximum
20	1	434.27	20.98	114.90	39.0	368.0	467.8	505.2	601.8
	3	484.83	34.31	176.92	9.8	409.1	470.2	565.4	877.3
	5	382.75	32.3	188.83	71.0	282.7	327.5	453.8	868.9
mean for 20 min pressing		433.95							
30	1	411.65	34.31	187.89	16.8	322.9	456.0	542.5	784.0
	3	372.09	20.22	110.74	144.5	271.2	418.5	445.6	542.7
	5	395.29	25.97	142.25	185.1	268.1	374.9	504.7	698.5
mean for 30 min pressing		393.01							
40	1	335.01	18.33	100.42	144.6	258.1	338.1	428.8	484.5
	3	429.56	30.31	166.02	81.4	357.1	451.8	575.1	715.6
	5	489.71	27.92	152.91	224.6	362.2	459.9	620.9	849.1
mean for 40 min pressing		418.09							

## Data Availability

The original contributions presented in this study are included in the article. Further inquiries can be directed to the corresponding author.
